# Associations between social connections, their interactions, and obesity differ by gender: A population-based, cross-sectional analysis of the Canadian Longitudinal Study on Aging

**DOI:** 10.1371/journal.pone.0235977

**Published:** 2020-07-30

**Authors:** Zeinab Hosseini, Gerry Veenstra, Nadia A. Khan, Annalijn I. Conklin

**Affiliations:** 1 Collaboration for Outcomes Research and Evaluation, Faculty of Pharmaceutical Sciences, University of British Columbia, Vancouver, Canada; 2 Department of Sociology, University of British Columbia, Vancouver, Canada; 3 Department of Medicine, University of British Columbia, Vancouver, Canada; 4 Centre for Health Evaluation and Outcome Sciences, St. Paul’s Hospital, Vancouver, Canada; East Tennessee State University, UNITED STATES

## Abstract

**Objectives:**

To quantify the link between four different types of social ties and objective measures of abdominal and general obesity, and to explore their inter-relationships in association with obesity using a gender-sensitive analysis.

**Methods:**

A cross-sectional analysis of 28,238 adults (45–85 years) from the baseline Canadian Longitudinal Study on Aging Comprehensive cohort (2012–15). Social ties (marital status, living arrangement, social network size, and social participation) and measured anthropometry (body mass index, waist circumference) were analyzed using linear and logistic regression models with interaction terms conditioned on known confounders.

**Results:**

We found that being single, widowed or divorced/separated was associated with worse anthropometric outcomes in women, including higher odds of both abdominal and general obesity, and that associations were enhanced when combined with limited social participation, lone-living and greater social network size. Few clear associations were observed in men. Limited social participation (no social activities at least once/month) among women was associated with larger waist circumference (+4.19 cm [95% CI: 1.86, 6.52]) and higher odds of both abdominal and general obesity. By contrast, associations appeared to be reversed in men: lone-living and smaller social networks were associated with lower odds of obesity, compared to co-living and larger social networks. We also found that more regular social participation can potentially mitigate the adverse associations between non-partnership (single, divorced) and obesity in women. Overall, the combined influence of two types of social tie deficits on excess weight measures was more pronounced in women than men.

**Conclusions:**

Results highlight the importance of considering how the role of social ties for obesity prevention may differ for women and men. Frequent social participation and number of social contacts may matter for assessing whether divorced, single or lone-living older women are at risk of obesity while living arrangement and social contacts may matter for obesity in men.

## Introduction

Healthy aging across the lifespan is an important public health and policy issue globally, including in Canada [1]. In fact, the third Sustainable Development Goal (SDG) sets out good health and wellbeing as a top milestone for governments to achieve. Yet large systematic disparities in health exist within, and between, countries—disparities that are linked to degrees of social disadvantage and wealth [2]. The burden of chronic conditions such as obesity has doubled in Canada since the 1970s and, while obesity is more common in men of all ages, women have more extreme classes of obesity that have the fastest rates of increase [3,4]. About 27% of Canadian adults have obesity, but the burden is higher among over-60s (33%) and among maritime provinces (e.g. 40% in Newfoundland and Labrador) [5,6]. Canada’s government and other states have a duty to close the gap in avoidable and unfair differences in health by tackling upstream social determinants that are known to drive health inequities. Understanding which social factors are needed for equitable health and wellbeing is critical to designing appropriate public health strategies and supporting government actions to reduce health inequities.

Discrimination (particularly gender) and poor social resources are both important upstream determinants of health [7], yet little research examines their intersections or their interactions. Lack of social connections can affect morbidity and mortality to a greater magnitude than smoking and other conventional risk factors [8,9]. But, this link will likely differ by gender because older women and men vary in the number and sizes of their different ties [10] and in their health impacts [11]. Half of over-80 Canadians live alone [12], but more senior women (31.5%) live alone than do senior men (16%) [13]. For example, more women (16%) than men (13%) aged 55 and older are divorced or separated [14]. Notably, different social meanings are attributed to being partnered, single widowed or divorced/separated for older women and men, which have different resource implications for divisions of labor and access to resources, and preventive behaviours [15,16]. Since gender is a socio-cultural construct that fundamentally shapes the social and economic conditions of women and men and the availability of valued resources and opportunities important for health [7,17], gender discrimination is a significant upstream determinant of obesity that deserves more commensurate research attention [18,19]. Gender differences and social inequalities also intersect with aging to create unique social conditions that can lead to health inequities and thus specific attention to women in older age groups is needed to achieve healthy aging by promoting social environments [20,21].

Obesity has been shown to be linked to social connections, and could be one of the biological pathways for the established link between social connections and mortality [8,9,22]. However, studies of obesity have focused more on the functional aspects of ties such as marital quality or perceived social support [23,24], or have combined measures of support with marital status and other structural ties [25–27]. Some studies of marital status and obesity have indicated that non-married women have higher odds of obesity than married women [28–30], whereas other research showed that both married women and men were more likely to have obesity than non-married adults [23,31,32]. Few studies have considered other structural ties (e.g. living arrangement, social network size) and obesity [8,23,24.^33^]. Thus, there are several gaps in the evidence because current research is rarely gender-specific, combines multiple different ties into an index, or collapses multiple categories into non-specific binary exposures [27,34–36]. These knowledge gaps limit effective targeting of obesity prevention.

In addition, the many different types of relationships that might influence obesity have not been examined for their joint effects [22,27]. The different configurations of older women’s and older men’s social contexts are an important source of heterogeneity that could be linked to obesity in unique ways. For example, widowhood may be linked to obesity to a greater extent for older individuals who have limited social engagement. Intersectionality Theory [21,37,38] suggests that the absence of different types of social resources would create a double burden and that the influence of diverse combinations of two social factors is not fully described by the independent effects of single factors (e.g. marital status, social participation). This synergistic notion was shown for widowed older adults in the UK whose diet quality was worse when lone-living or their contact with friends was infrequent [39]. We therefore aimed to provide new evidence on the synergistic influence on obesity of multiple social ties.

Using a new population-based aging cohort in Canada, we assessed whether four different types of social ties were related to two measured anthropometric outcomes in gender-specific ways, and whether different social connections interacted to produce a combined effect on obesity. We hypothesised that each social tie variable would be linked to obesity, with stronger effects of marital status and living arrangement in men and social engagement in women. We further hypothesised that frequent social activities or co-living might mitigate the negative association of being single or widowed or having rare social contact with obesity. Similarly, frequent social contact may lessen the negative association of living alone with obesity.

## Methods

### Study design and population

We used the baseline data (2012–15) of the population-based Canadian Longitudinal Study on Aging (CLSA) Comprehensive cohort that provided information on physical, social and psychological factors as well as objective measures of abdominal and general obesity from approximately 30,000 respondents aged 45–85 years living in the community [40]. Excluded from CLSA Comprehensive cohort were people not speaking English or French; having cognitive impairment; with temporary visa or transitional health coverage; living in the three territories, some remote regions, on federal First Nations reserves and other First Nations settlements in the provinces, Canadian Armed Forces full-time members, and living in institutions; and participants who did not give anthropometrics [41]. Our available sample included participants with complete data on structural social ties, covariables, and anthropometry (n = 28,238). All participants gave written informed consent and the study was approved by the University of British Columbia Behavioural Research Ethics Board (H19-00971).

### Anthropometric outcomes

Clinically measured anthropometry included height (m), weight (Kg) and waist circumference (WC), measured from halfway between the last rib and the iliac crest bone [41]. We used WC to classify participants as having abdominal obesity. Thus, males with WC ≥ 102 cm and females with WC ≥ 88 were considered as abdominally obese [42] or not. We calculated body mass index (BMI) by dividing weight with height squared, and then dichotomised BMI to identify participants who had general obesity. A cut-off of BMI ≥ 30 kg/m^2^ was considered for being generally obese in both women and men. Both continuous (BMI, WC) and binary (abdominal and general obesity) outcomes were analysed.

### Structural types of social ties

Structural aspects of social ties were assessed by marital status (partnered (as reference), single, widowed, divorced/separated), living arrangement (lone-living, co-living (as reference)), social network size (sum of all social contacts) and social participation (regular social activities) [8]. Social network size (range: 1–573) was measured by summing responses to eight questions on the known number of children, siblings, relatives, close friends, neighbours, work/school colleagues, community and people known through other activities; with levels defined as quartiles (highest Q4, those with the largest social network size, were the reference). Social participation was assessed similar to other CLSA research [43] based on questions about frequent participation (at least once a month or more often versus less than once a month) in social activities in the last year across eight settings (family, religious, sports, educational, social clubs, charity, neighbourhood and other recreational) [44]. The total sum of all frequent social activities contributed to a novel social participation measure (range:0 to 8). Due to low numbers in the highest levels, we re-classified responses to have meaningful groupings for analysis: ‘none’ (0 regular activities), ‘a few’ (1–2), ‘some’ (3–4), and ‘a lot’ (5 or more) (reference).

### Covariables

Self-reported confounders were age and age squared, smoking status (ever/never), province and education (less than secondary school; secondary school; some post-secondary education including degree/diploma; university degree). Our selection of covariables was informed by existing literature on factors known to correlate with our exposure and outcome. As known confounders, each is associated with social relationships and independently with obesity [9,45–47].

Sensitivity analyses included other factors that may be additional confounders or are sex-specific biological determinants of obesity [23,24,33]: Indigenous status (Y/N)[21]; lifestyle factors (e.g. weekly alcohol intake; daily servings of fruits and vegetables; sleep duration and quality; amount of daily physical activity [48]); psychosocial factors (Center for Epidemiological Studies Depression scale (CESD) [49], life satisfaction and depression medication); chronic conditions (hyper-and hypothyroidism; rheumatoid arthritis; asthma; cardiovascular disease; cancer, osteoporosis; diabetes; Parkinson's; and stroke; or relevant medications); and self-rated health. Sex-specific risk factors related to women’s reproductive status were assessed by number of biological children (continuous), menopause status (Y/N) and hormone replacement therapy use (ever/never). Self-reported male/female was used as an effect modifier to indicate gender-based differences in the relationships investigated.

### Statistical analysis

Means (SD) and frequencies were used to describe the characteristics of the cohort across levels of four structural social ties. The CLSA Comprehensive cohort only included the Canadian population that resided within 25 km of the Data Collection Sites, hence survey weights were not used as this sample was not nationally representative [41]. We used Cramer's V test to assess the relationship between each pair of social tie variables (e.g. marital status & social participation level) [50].

Objective one aimed to examine the gender-specific main and independent (of other ties) associations between each social tie and each anthropometric outcome. For this objective we ran a series of multivariable linear (WC and BMI as outcomes) and logistic (central and general obesity as outcomes) regression models. First, we assessed main associations with mutual adjustment for age, age squared, education, smoking and province. Second, we additionally adjusted for all other social tie variables so that the regression results could be interpreted as the independent association of each social tie with each outcome. Each model included an interaction term between the social exposure and self-reported female/male, giving gender-specific results. We used reverse coding of the interaction term (female = 0 and male = 1; female = 1 and male = 0) to provide estimates for each reference group from the same model. We reported results as post-estimated adjusted means, or odds ratios (OR), and 95% confidence intervals (95% CI). Sensitivity analyses tested the robustness of the independent associations to additional confounding.

Objective two considered whether the association between each social tie and obesity changed when a second social tie was considered (joint association). For example, the association of marital status and obesity may be stronger or weaker for those that have no social participation compared to those that have the most social participation. For Objective two, WC and BMI were analysed using gender-stratified multivariable linear regression models conditioned on key confounders with interaction terms for each pair (e.g., marital status by living arrangement) and reported as adjusted means and 95% CI. All analyses were performed using Stata/SE 14 (StataCorp LP, College Station, TX).

## Results

The mean age of participants was 63 (SD 10) years with 50.6% women (S1 Table). A higher proportion of women were widowed, lone-living, had a smaller social network size, and participated in more social activities compared to men (Table 1). The majority (91%) reported either good or excellent health with 35.5% of women and 28% percent of men being non-smokers (Table 1). Fewer women (41%) than men (51%) had the highest education level of at least a university degree. Gender-based differences across social tie categories were seen in mean age, education level, and smoking status (Table 1). Average levels of each outcome also differed between and within the four social tie variables, with patterns distinct to each exposure and outcome for women and men (Table 1). There was a strong association between marital status and living arrangement (Cramer's V = 0.77) and weak relationships between all other pairs of social ties.

**Table 1:** Descriptive characteristics across structural social ties among older women and men in the CLSA (2012–15).

	Mean (SD) age	Highest education level %	Non-smoker %	Mean (SD) WC (cm)	Abdominal obesity %	Mean (SD) BMI (kg/m^2^)	General obesity %
**Women**							
**Total (14,289)**	62.6 (10.2)	40.9	35.5	119.6 (17.2)	46.9	27.8 (5.9)	28.8
**Marital status** *							
Partnered	60.6 (9.4)	43.6	37.1	118.2 (16.6)	43.4	27.4 (5.6)	26.4
Single	60.2 (9.8)	49.9	34.6	118.9 (17.3)	51.6	28.9 (6.8)	34.5
Widowed	73.0 (8.5)	26.0	35.0	126.3 (17.8)	53.7	28.0 (5.8)	31.2
Divorced	62.8 (9.3)	37.9	30.9	119.9 (17.8)	51.3	28.3 (6.4)	32.8
**Living arrangement**							
Co-living	60.5 (9.5)	42.7	36.6	118.3 (16.7)	44.7	27.6 (5.8)	27.6
Lone-living	67.8 (9.9)	36.2	32.9	123.0 (18.0)	52.3	28.3 (6.2)	32.0
**Social network size** ^†^							
Largest, Q4 (220–573)	60.1 (9.7)	51.9	38.4	117.9 (17.0)	45.7	27.9 (6.0)	29.4
Q3 (146–219)	60.9 (9.8)	45.1	35.5	118.7 (17.2)	46.0	27.8 (5.8)	28.3
Q2 (86–145)	63.3 (9.9)	37.4	35.6	119.6 (17.0)	46.2	27.7 (5.8)	28.4
Smallest, Q1 (1–85)	65.8 (10.3)	30.2	32.8	122.1 (17.4)	49.5	27.8 (5.9)	29.3
**Social participation**^‡^							
A lot (5–8)	63.7 (10.2)	48.8	40.2	119.6 (17.1)	45.5	27.6 (5.7)	27.2
Some (3–4)	61.5 (10.0)	38.2	33.2	119.1 (17.2)	45.6	27.7 (5.8)	28.0
A few (1–2)	62.0 (10.2)	25.8	28.3	120.9 (17.4)	53.7	28.7 (6.5)	35.1
None (0)	62.7 (10.3)	19.4	26.5	122.0 (18.2)	57.7	29.3 (6.8)	40.6
**Men**							
**Total (13,949)**	63.0 (10.2)	50.7	28.0	122.4 (15.9)	40.8	28.3 (4.7)	29.7
**Marital status** *							
Partnered	62.8 (10.1)	52.5	28.7	122.3 (15.6)	40.2	28.3 (4.7)	29.3
Single	59.0 (9.1)	46.3	31.5	121.3 (16.1)	41.4	28.2 (5.6)	32.1
Widowed	73.3 (8.6)	39.5	21.4	125.9 (18.1)	46.3	28.1 (4.7)	28.1
Divorced / Separated	62.7 (9.6)	44.1	22.3	122.5 (17.2)	42.9	28.2 (4.7)	31.5
**Living arrangement**							
Co-living	62.5 (10.1)	52.0	28.6	122.3 (15.7)	40.2	28.3 (4.7)	29.5
Lone-living	65.6 (10.4)	43.4	24.7	123.3 (17.2)	44.1	28.1 (5.0)	30.6
**Social network size** ^†^							
Largest, Q4 (220–573)	61.2 (9.8)	56.5	29.9	122.3 (15.6)	43.4	28.8 (4.7)	33.9
Q3 (146–219)	61.6 (10.0)	53.3	29.2	122.0 (15.6)	39.6	28.3 (4.8)	30.1
Q2 (86–145)	63.6 (10.2)	49.2	27.3	122.5 (15.8)	39.7	28.0 (4.6)	26.9
Smallest, Q1 (1–85)	65.8 (10.3)	43.1	25.5	122.9 (16.7)	40.3	27.9 (4.8)	27.2
**Social participation**^‡^							
A lot (5–8)	64.1 (10.4)	59.4	40.2	122.5 (15.8)	41.2	28.4 (5.5)	29.5
Some (3–4)	62.1 (10.0)	49.4	28.0	122.2 (15.6)	39.2	28.1 (5.7)	29.0
A few (1–2)	62.9 (10.2)	38.1	24.5	122.8 (16.7)	43.1	28.4 (5.2)	30.9
None (0)	63.1 (10.5)	26.3	21.1	122.9 (17.4)	47.0	28.6 (5.8)	33.9

BMI, body mass index; CLSA, Canadian Longitudinal Study on Aging; WC, waist circumference. Abdominal obesity cut-off: males, WC ≥ 102 cm; females, WC ≥ 88. General obesity cut-off: BMI ≥30 kg/m2. The highest education level: at least a university Bachelor’s degree.

*Partnered included married or living as married; divorced includes separated.

^†^Social network size (1–573) was a sum of responses to eight questions about the number of social contacts the respondent knows (e.g. siblings, children, colleagues, etc), with network size increasing from smallest (Q1) to largest (Q4).

^‡^Social participation was a sum of responses to eight questions about regular (≥ once per month) participation in different social activities, that was re-classified into four levels of social participation, that was re-classified into four levels of social participation (5 or more, ‘a lot’, as reference).

### Gender-specific associations between structural social ties and obesity

**Fig 1:**
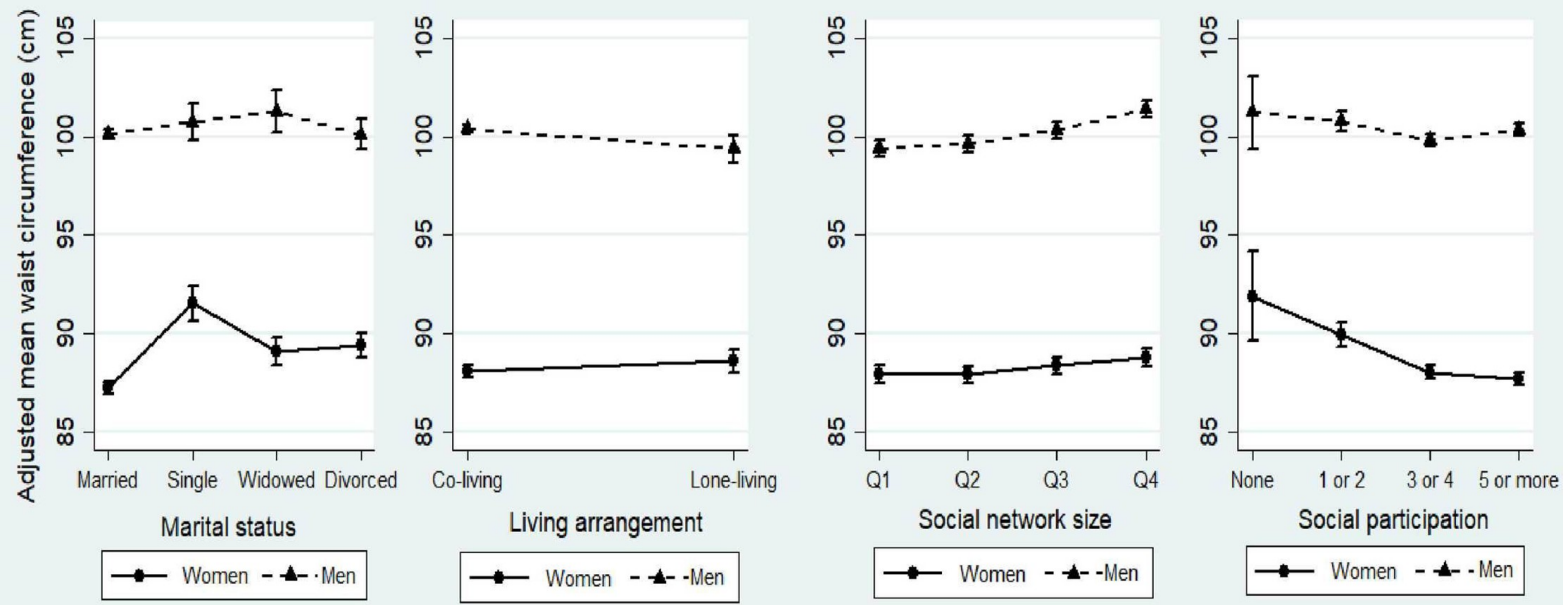
Adjusted mean waist circumference across levels/categories of four structural social ties among older women and men in the CLSA (2012–15). Women, solid line; men, dashed line. Partnered was married or living as married; divorced includes separated. Social network size (1–573) was a sum of responses to eight questions about the number of social contacts the respondent knows (e.g. siblings, children, colleagues, etc), with network size increasing from smallest (Q1) to largest (Q4) (total number of contacts in each quartile: Q1:1–85, Q2:86–145, Q3:146–219, Q4:220–573). Social participation was a sum of responses to eight questions about regular (≥ once per month) participation in different social activities that was re-classified into four levels of social participation (0 (none), 1–2 (a few), 3–4 (some), 5–8 (a lot)).

In multivariable-adjusted models, we found significant independent associations between each social tie and adjusted mean WC in both women and men, but with gender-specific patterns (Fig 1). Women who were single, lone-living, had the largest network size or had no social activities had the highest adjusted mean WC. Regression coefficients in S2 Table showed that the largest unit differences in WC in women were among those who were single (+4.32 cm [95% CI: 3.34, 5.29]) or had no social participation (+4.19 cm [1.86,6.52]), compared to partnered women or women with high social participation. By contrast, adjusted mean WC was highest among men who were widowed, co-living, and had the largest network size (Fig 1). Regression coefficients showed that the largest unit difference in WC in men was observed for social network size: men in the lowest quartile (Q1) had lower WC (-2.02 cm [-2.64, -1.40]) compared to men in the highest quartile (S2 Table). In both women and men, adjusted mean levels of BMI across all social ties mirrored the patterns of WC but with smaller effect sizes (S1 Fig).

Using clinically meaningful outcomes, we found that all non-partnered categories and limited social activities (≤2) were each independently linked to higher odds of abdominal obesity (OR range: 1.25–1.53) and general obesity (OR range: 1.36–1.69) in women, compared to partnered or socially active counterparts (Table 2). Women who were single and had no social participation had the strongest associations with abdominal and general obesity. We found no association between living arrangement and both clinical outcomes in women. By contrast for men, only the single category was associated with higher odds of abdominal obesity (1.18 [1.02, 1.37]) compared to partnered men. Moreover, lone-living men had lower odds of general obesity (0.85 [0.75, 0.97]), and those with small social networks also had lower odds of both abdominal (0.76 [0.69, 0.84]) and general obesity (0.69 [0.61, 0.76]), compared to counterparts (Table 2).

**Table 2:** Independent association between structural social ties and odds of general and abdominal obesity among older women and men in CLSA (2012–15).

	Abdominal obesity		General obesity	
	Women	Men	Women	Men
**Marital status**				
Partnered*	Ref	Ref	Ref	Ref
Single	**1.53 (1.33, 1.75)** ^§^^‖^	**1.18 (1.02,1.37)** ^†^^‖^	**1.63 (1.41,1.88)** ^§^^‖^	1.16 (0.99,1.36) ^‖^
Widowed	**1.25 (1.1,1.42)** ^§^	1.14 (0.95,1.36)	**1.42 (1.24,1.63)** ^§^^‖^	1.09 (0.9,1.32) ^‖^
Divorced/ separated	**1.33 (1.19,1.48)** ^§^^‖^	1.10 (0.96,1.26) ^‖^	**1.38 (1.23,1.55)** ^§^^‖^	1.09 (0.95,1.26) ^‖^
**Living arrangement**				
Co-living	Ref	Ref	Ref	Ref
Lone-living	1.02 (0.92,1.13)	0.92 (0.82,1.04)	1.06 (0.95,1.19) ^‖^	**0.85 (0.75,0.97)** ^**†**^^**‖**^
**Social network size (quartile)**				
Largest, Q4 (220–573)	Ref	Ref	Ref	Ref
Q3 (146–219)	0.96 (0.87,1.06) ^‖^	**0.85 (0.77,0.94)** ^‡^^‖^	0.91 (0.82,1.01)	**0.83 (0.75,0.92)** ^‡^
Q2 (86–145)	**0.90 (0.81,0.99)** ^†^	**0.81 (0.73,0.89)** ^§^	**0.89 (0.8,0.99)** ^†^^‖^	**0.70 (0.63,0.78)** ^‡^^‖^
Smallest, Q1 (1–85)	0.93 (0.84,1.03) ^‖^	**0.76 (0.69,0.84)** ^§^^‖^	0.90 (0.81,1.01) ^‖^	**0.69 (0.61,0.76)** ^‡^^‖^
**Social participation**				
A lot (5–8)	Ref	Ref	Ref	Ref
Some (3–4)	1.02 (0.94,1.1)	0.93 (0.86, 1.01)	1.03 (0.94,1.11)	0.96 (0.88,1.04)
A few (1–2)	**1.31 (1.18,1.45)** ^§^^‖^	1.04 (0.94, 1.15) ^‖^	**1.36 (1.22,1.52)** ^§^^‖^	1.01 (0.91,1.13) ^‖^
None (0)	**1.46 (1.07,1.99)** ^†^	1.11 (0.85, 1.45)	**1.69 (1.24,2.32)** ^‡^^‖^	1.06 (0.8,1.41) ^‖^

Abdominal obesity cut-off: males, WC ≥ 102 cm; females, WC ≥ 88. General obesity cut-off: BMI ≥30 kg/m2. Gender-specific odds ratio (CI95) obtained by multivariable logistic regression analysis of sample (N  =  28,238) using an interaction term between each structural social tie and gender adjusted for age, age2, education, smoking, province and other ties.

* Partnered was married or living as married; divorced includes separated; Social network size (1–573) was a sum of responses to eight questions about the number of social contacts the respondent knows (e.g. siblings, children, colleagues, etc.), with network size increasing from lowest (Q1) to highest (Q4). Social participation was a sum of responses to eight questions about regular (≥ once per month) participation in different social activities that was re-classified into four levels of social participation (0 (none), 1–2 (a few), 3–4 (some), 5–8 (a lot)).

^†^ p < 0.05

^‡^ p < 0.01

^§^ p < 0.001

^‖^p for interaction < 0.05.

Sensitivity analysis including health behaviours, psychological factors and chronic conditions minimally attenuated most results and amplified some (e.g., social participation and anthropometric outcomes in women; social network size and WC and BMI in both). Two associations with WC in men lost statistical significance (marital status after including psychological factors, and living arrangement after including health behaviours), and adjusting for physical activity reversed and strengthened the association of social participation with WC in men.

### Gender differences in the inter-relations of marital status and other ties with anthropometric outcomes

**Fig 2:**
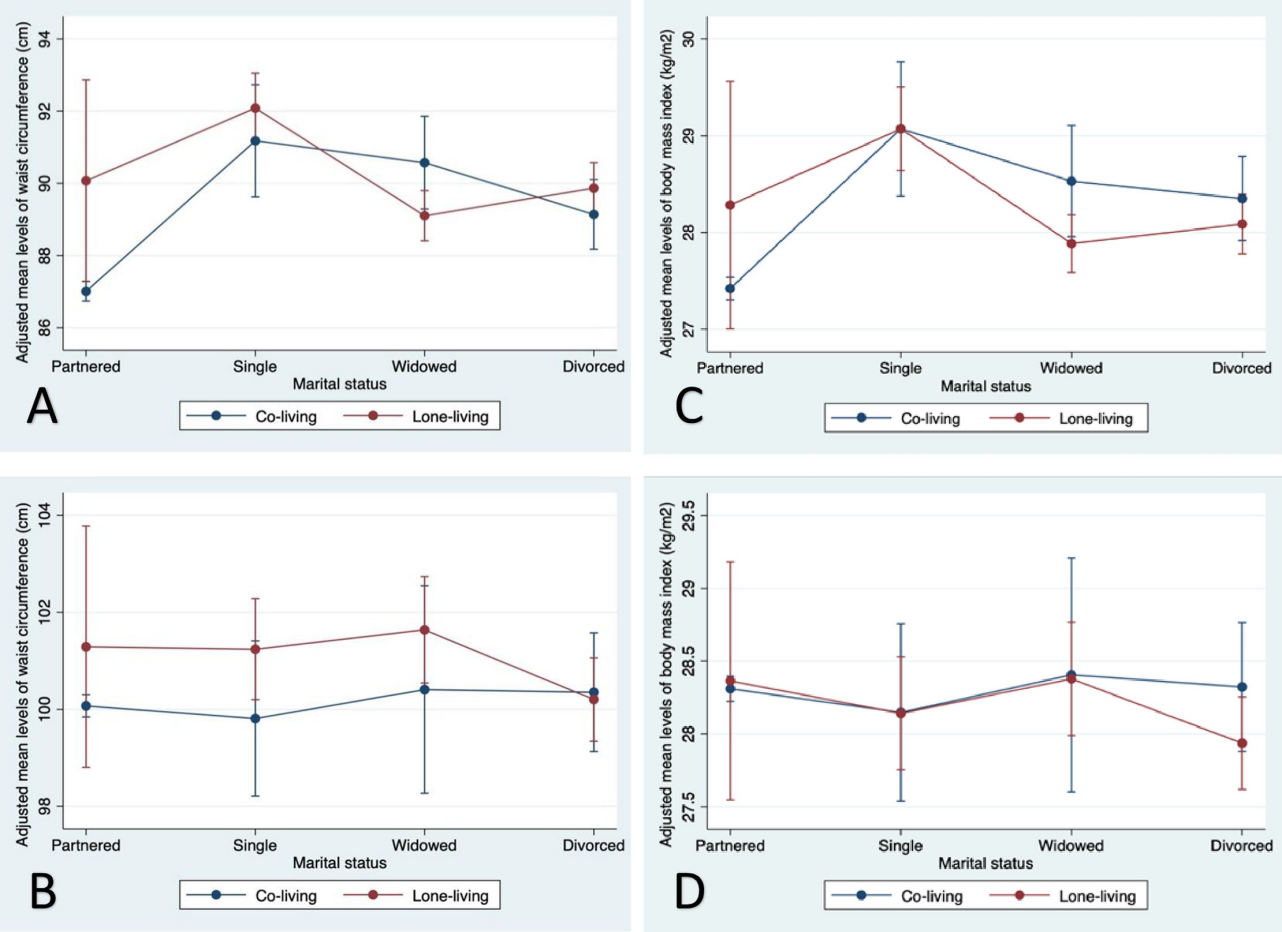
Adjusted mean waist circumference (Panel A, women; Panel B, men) and body mass index (Panel C, women; Panel D, men) across marital status categories by living arrangement among older women and men in the Canadian Longitudinal Study on Aging (2012–15). Partnered was married or living as married; divorced includes separated.

**Fig 3:**
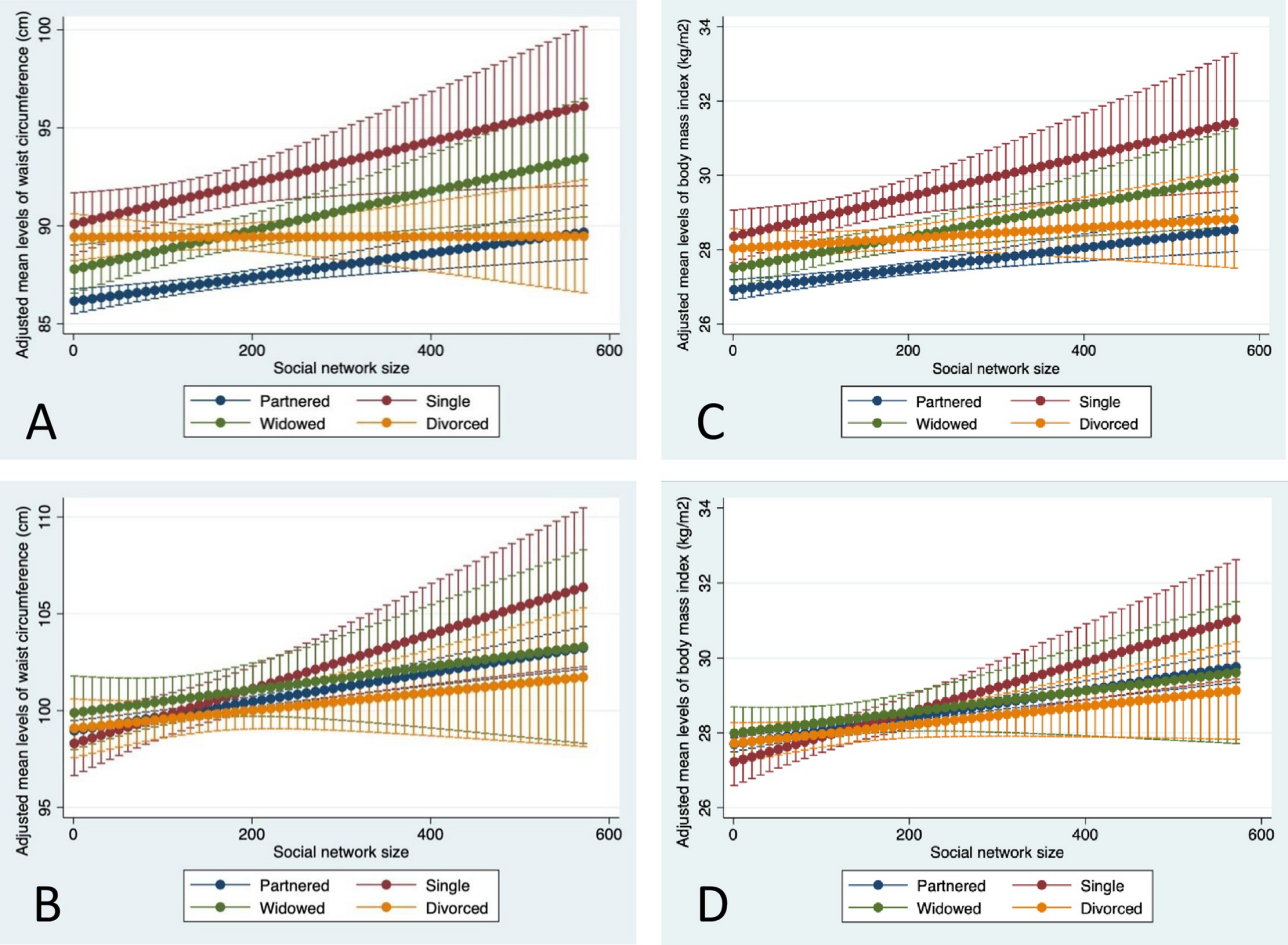
Adjusted mean waist circumference (Panel A, women; Panel B, men) and body mass index (Panel C, women; Panel D, men) across social network size, by marital status categories, among older women and men in the Canadian Longitudinal Study on Aging (2012–15). Partnered was married or living as married; divorced includes separated. Social network size (1–573) was a sum of responses to eight questions about the number of social contacts the respondent knows (e.g. siblings, children, colleagues, etc.).

**Fig 4:**
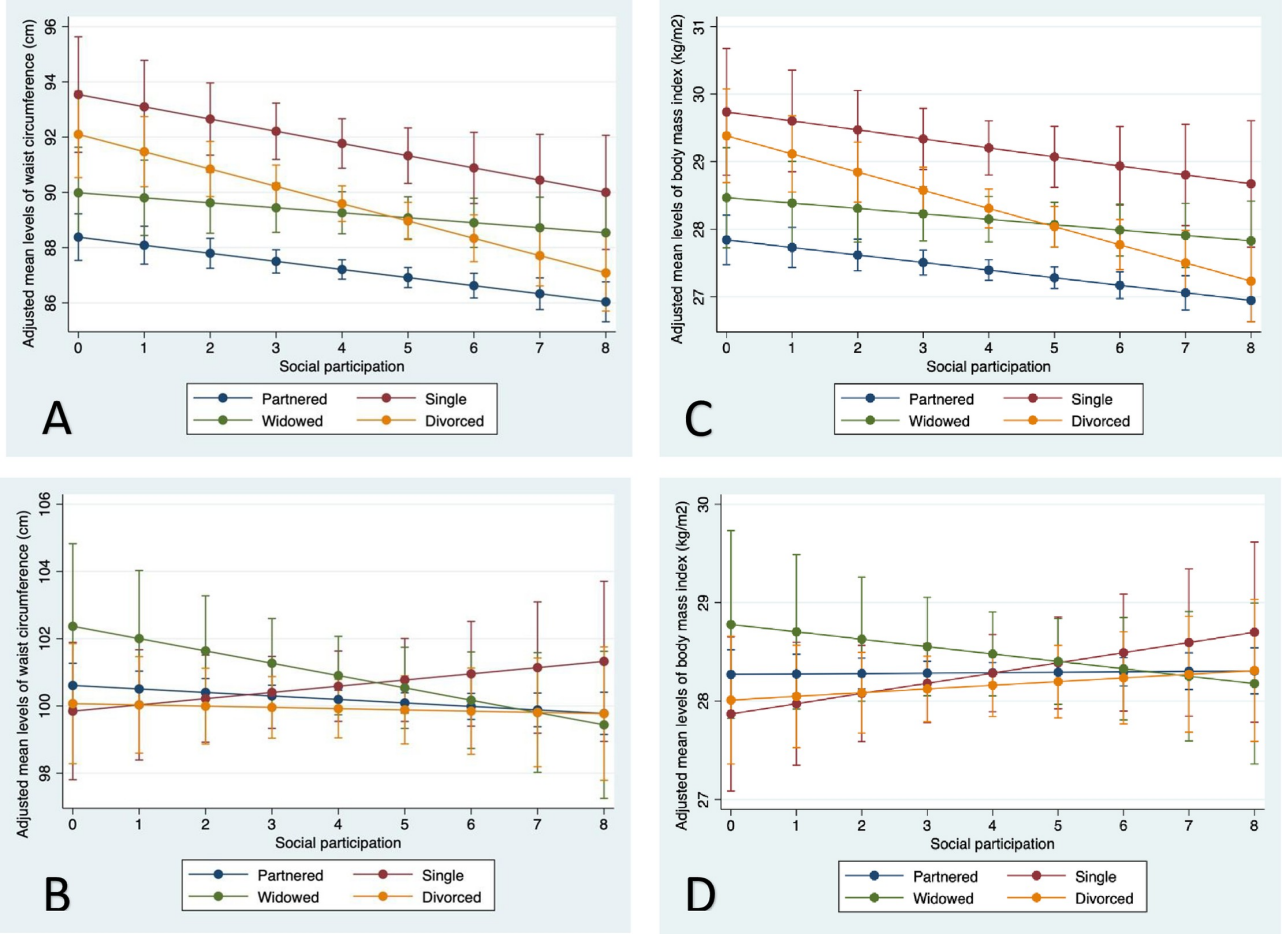
Adjusted mean waist circumference (Panel A, women; Panel B, men) and body mass index (Panel C, women; Panel D, men) across levels of social participation, by marital status categories, among older women and men in the Canadian Longitudinal Study on Aging (2012–15). Partnered was married or living as married; divorced includes separated. Social participation was a sum of responses to eight questions about regular (≥ once per month) participation in different social activities.

Figs 2–4 (S3 Table and S4 Table) showed how associations between marital status and WC and BMI were altered by living arrangement, social network size and social participation. All non-partnered women who were co-living had higher WC than partnered women who were co-living (Fig 2, panel A). The contrast in living arrangement for widowed women showed an opposing pattern to the contrast in living arrangement for all other marital status categories: lone-living widowed women had a lower WC (-0.97 cm [-3.85, 1.91]), compared to lone-living partnered, that was significantly different (p-interaction = 0.005) from co-living widowed women (+3.57 cm [2.26, 4.88]) relative to co-living partnered women. In men, with the exception of divorced/separated, we found higher mean WC in lone-living than co-living men with different marital statuses (Fig 2, panel B). Parallel findings were observed for BMI in women, whereas men’s BMI was higher only for divorced co-living men compared to lone-living counterparts (S3 Table and Fig 2, panel C and D).

All marital status categories had higher mean levels of WC with increasing size of social networks for women, with the exception of divorced women who did not differ in mean WC across quartiles of social network size. Also, single and widowed women had higher WC compared to partnered women across all social network size levels, especially at greater sizes, and differences in WC between divorced and partnered women were only observed in the lowest quartile (Fig 3, panel A). Differences between marital status categories in WC across social network size were less clear in men, with higher WC in single men having a network size of 250 or more contacts than divorced men with similar network sizes (Fig 3, panel B). BMI showed similar patterns to WC in women and men (Fig 3, panels C and D).

We found a consistent pattern for women in the joint association of marital status and social participation with WC. Non-partnered women who participated in two or fewer social activities had much higher mean levels of WC than partnered women with limited social participation, but these differences in marital status were reduced or removed for divorced women with the most social participation (Fig 4, panel A). The protective association of social participation was most notable for divorced women for whom mean WC converged with partnered counterparts. Nevertheless, both single and widowed women with 8 social activities had significantly higher WC than partnered counterparts (Fig 4, panel A). The role of social participation in mitigating the health-harming influence of non-partnered status on WC in men was less evident, although greater social participation appeared to be supportive for widowed men’s WC (Fig 4, panel B). We found similar patterns for BMI in both genders (Fig 4, panel C and D, and S4 Table).

### Gender differences in social network size and anthropometry, by social participation and living arrangement

**Fig 5:**
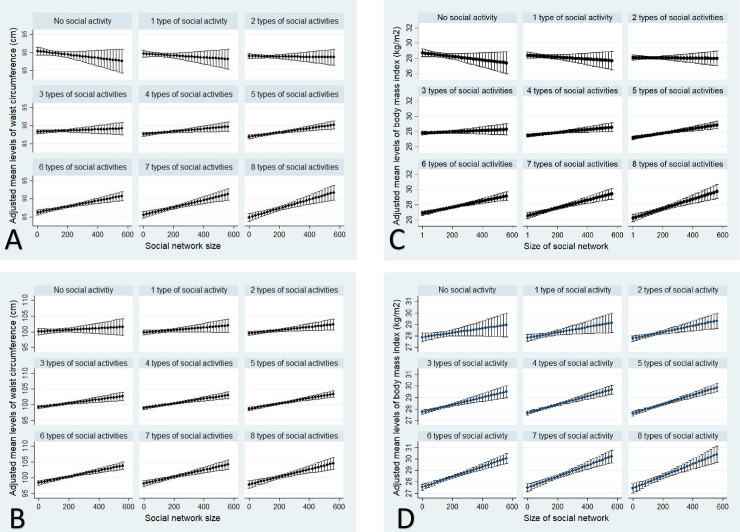
Adjusted mean waist circumference (Panel A, women; Panel B, men) and body mass index (Panel C, women; Panel D, men) across social network size, by level of social participation, among older women and men in the Canadian Longitudinal Study on Aging (2012–15). Social network size (1–573) was a sum of responses to eight questions about the number of social contacts the respondent knows (e.g. siblings, children, colleagues, etc. Social participation was a sum of responses to eight questions about regular (≥ once per month) participation in different social activities.

**Fig 6:**
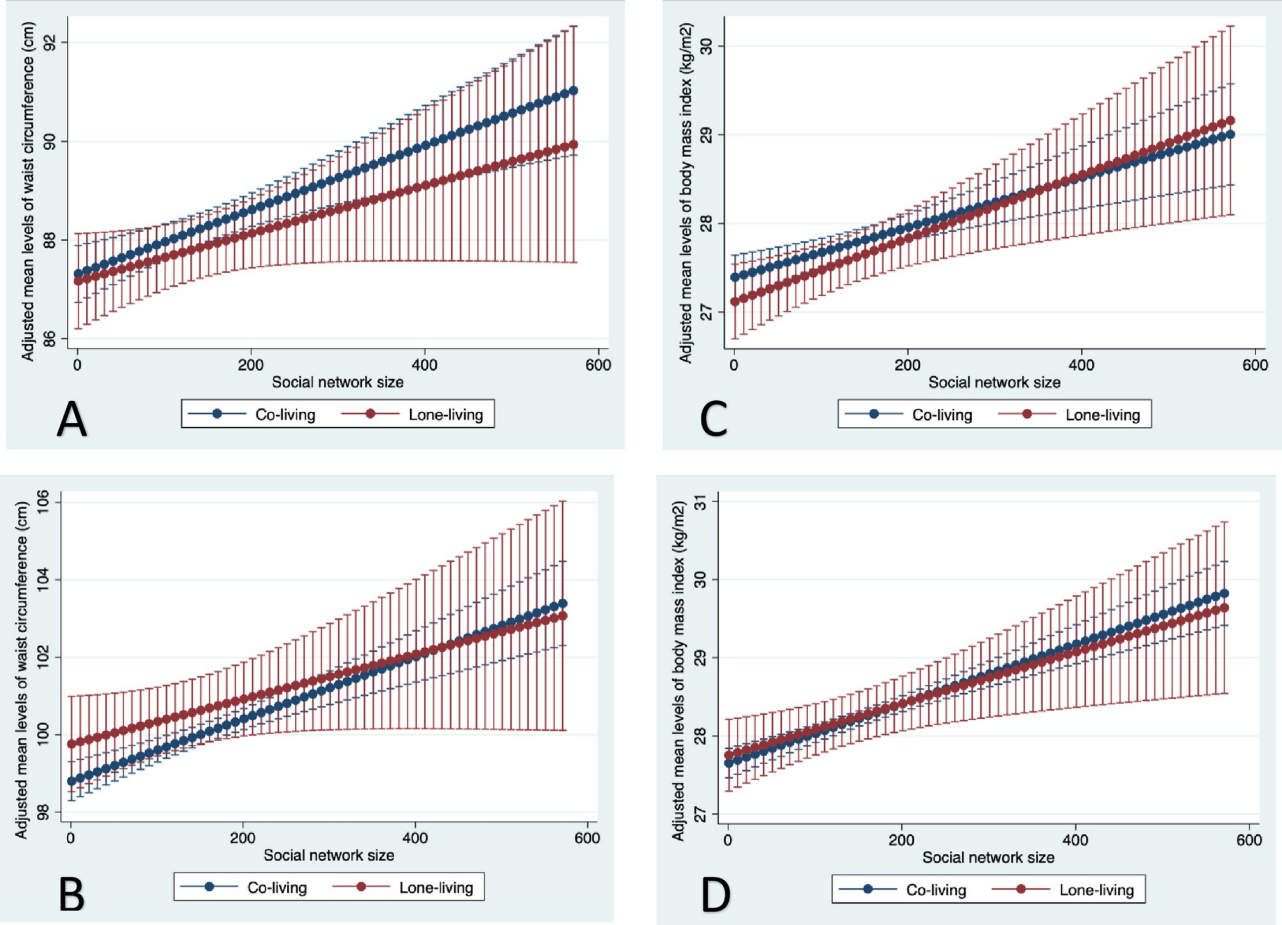
Adjusted mean waist circumference (women, panel A; men, panel B) and body mass index (women, panel C; men, panel D) across social network size, by living arrangement, among older women and men in the Canadian Longitudinal Study on Aging (2012–15). Social network size (1–573) was a sum of responses to eight questions about the number of social contacts the respondent knows (e.g. siblings, children, colleagues, etc).

Overall, we found that the independent association between social network size and WC varied by levels of social participation with gender-specific patterning. In women, mean levels of WC were inversely associated with social network size when social participation was limited or absent, but were positively associated when social participation involved five or more activities (Fig 5, panel A, and S5 Table). We found the lowest mean WC (84.9 [83.9–86.0]) occurred in women who participated in 8 activities but had only one social contact. By contrast, in men, we only observed a positive association between social network size and mean WC when they participated in three or more activities (Fig 5, panel B). We did not find any effect modification of the association between social network size and WC by living arrangement in either women or men (S6 Table and Fig 6, panels A and B). The patterns of BMI were similar to WC in both women and men (Fig 6 panels C and D, and S6 Table).

### Gender differences in social participation and anthropometry, by living arrangement

The independent inverse association between social participation and WC was similar for co-living and lone-living women and men (S2 Fig, and S6 Table). With the exception of WC in men, the lowest mean levels of WC and BMI were observed among adults who were lone-living and highly socially active (S2 Fig).

## Discussion

This population-based, cross-sectional study of nearly 30,000 adult Canadians used a gender-sensitive analysis to assess the relative contribution, and inter-relations, of different types of social ties on several anthropometric outcomes for women and men. We showed clear gender differences in the magnitude and direction of association, depending on the exposure and outcome measure. Generally, we found that non-partnership and less social participation were positively associated with WC in women, and smaller social network size and lone-living were inversely associated with WC in men.

Notably, stronger associations were seen for marital status and social participation in women and for social network size and living arrangement in men. These results only partly supported our first hypothesis that social engagement would be stronger for women and living arrangement stronger for men. We also found that larger social network size and more regular social participation can potentially mitigate the adverse relationship between non-partnership (single and/or widowed), and measures of obesity in women, which we hypothesized. Overall, the joint associations were more pronounced in women than men. Contrary to our hypothesis, co-living did not appear to mitigate the adverse relationship of limited social participation or lack of social contacts and obesity.

### Findings in the context of previous research

The existing literature provides mixed results for the link between social ties and obesity. Similar to our work, previous studies in US [28], Sweden [29] and Canada [30] have shown that widowed [28,30] and single [29] women are more likely to be obese (abdominally or generally) than married women. Although other studies from Iran [31], England [23] and Poland [32] reported that both married women and men had higher odds of obesity than non-married adults, the latter studies did not distinguish widowed status from single as they dichotomized marital status (i.e., married vs not married) [23,32] and/or they included a much younger population (from 15 to 65 years) [31,32].

Separate research examining social network size has shown a protective effect on mortality in older men, but that research used indices combining multiple ties such as marital status [51] and social participation [51,52] and even relationship quality [52]. Two cross-sectional studies of obesity show conflicting results. One cross-sectional study on English older adults did not find a link between any CVD risk factors including obesity to the number of children, relatives and friends the participants felt close to (results not gender-specific) [23], whereas another cross-sectional study of Korean older adults reported that higher social network size was linked to higher BMI in men only [33]. Our results showed that smaller social network sizes were related to more favourable anthropometric outcomes in men. Our results also indicated that participation in multiple social activities was linked to lower obesity which further concurs with other research [23,27]. Our findings were less notable regarding living arrangement and anthropometric outcomes among women and men as similarly reported in other studies [8,24].

Of significance, the inter-relations between different types of social ties revealed an important source of heterogeneity and unique associations with obesity in women and men. We found that social network and social participation can potentially mitigate the association between non-partnership and obesity outcomes in women. Little research has explored the joint association of social ties and health outcomes or behaviours, and there are none examining obesity outcomes. A 20-year study of 11,000 participants (aged 10 years and older) investigated marital status and living arrangement in women and men. The study found a higher risk of mortality in unmarried women who were co-living with dependent children compared to married women [53]. Interrelations of different structural social ties has also been assessed in relation to healthy behaviours, with living arrangement potentially mitigating the adverse association between widowhood and low vegetable variety [39]. That study found stronger associations in men than women, which is contrary to our findings. Overall, previous research has typically been limited by assessing multiple social ties not as singular measures but as composite scores that mask the relative contribution and inter-play of different ties [8,9].

Our findings revealed remarkable differences between women and men in the links between social ties and obesity, thus reinforcing how gender, a social-cultural factor, shapes how social ties impact health through physiological pathways [22]. In women, obesity measures were mainly related to marital status and social participation as opposed to men for whom social network size and living arrangement mattered most. Studies generally incorporate gender/sex as a control factor in their analysis [23,27,54]. The few studies assessing gender differences have reported mixed findings for significant interaction between social ties and gender [26,33,55].Similar to our results, two studies showed a strong association between social network size and BMI only in men [33]. However, other research did not report any significant differences between women and men in the association of a social isolation index with BMI [25]. Our study did not use an index of social isolation and so findings are not directly comparable. The more pronounced results for marital status in women, moreover, may be due to gender-specific social values attributed to marriage based on social expectations for parenthood or higher economic status which is achieved through partnership [24,56].

### Methodological considerations

We acknowledge a number of limitations of this study. The cross-sectional design limits causal inference although it is informative of potentially relevant relationships for prospective examination. Self-reported measures may introduce recall or survey effect bias in results. The social network size variable may have less variance in the sample than the population as the total number of social contacts for each setting could not be specified beyond 400. Moreover, social relationship measures were assessed as a state and so recent transitions such as widowhood or separation could have a greater influence on obesity than those marital states that occurred longer in the past. Despite not measuring gender self-identity, the self-reported male/female variable provides a highly correlated proxy to produce estimates for women and men that could reveal potential gendered power relations [57]; notably, other research suggests that 0.4% of American adults identify their gender different from their sex [58]. Although we included a broad range of covariables for sensitivity analyses, there is likely to be residual confounding from unobserved differences. Moreover, imprecise measurement of self-reported covariables such as physical activity may have introduced bias that would either attenuate or inflate observed associations [59,60], and thus might partly explain the amplified association that was reversed for social participation and WC in men. Finally, results are generalizable for middle- and older-age adults living in the community in high-income countries but not to other settings or younger populations.

This study had several strengths including the large sample size, adjustment for multiple known confounders and lifestyle variables, gender-specific estimation, effect modification and clinical measured anthropometric outcomes. We considered the main, independent and joint associations of separate measures of different relationships rather than using a summary index that masks the relative contribution of each type of connection. A particular strength of our study is in its consideration of the more complete picture of both gender and interactions of social relationships in relation to adiposity which has added nuances to the literature and helps to better inform more effective, and equitable, obesity prevention and healthy aging strategies.

## Conclusions

This study provides a more comprehensive and accurate picture of the intersecting and mutually reinforcing upstream determinants of obesity. We found a clear link between different types of social ties and obesity in Canadian adults, and the influence of social connections and their interactions on adiposity showed a differential pattern by gender. Future work needs to use longitudinal study designs to support causal inference and help inform potential structural interventions to tackle the growing burden of chronic disease and health inequities.

## Supporting information

S1 Table Descriptive characteristics across structural social ties among older adults in CLSA (2012–15). (DOCX)

S2 Table Independent associations between structural social ties and adiposity among older women and men in the CLSA (2012–15). (DOCX)

S3 Table Independent associations between marital status and adiposity, by living arrangement, among older women and men in the CLSA (2012–15). (DOCX)

S4 Table Independent association between marital status and measures of adiposity, by social participation or by social network size, among older adults in CLSA (2012–15). (DOCX)

S5 Table Independent association between lack of social participation and adiposity, by average social network size, among older women and men in the CLSA (2012–15). (DOCX)

S6 Table Independent association between living arrangement and adiposity, by social participation and social network size, among older women and men in CLSA (2012–15). (DOCX)

S1 Fig Adjusted mean body mass index with A, marital status, B, living arrangement, C, social participation and D, social network size among older women and men in the Canadian Longitudinal Study on Aging CLSA (2012–15). Women, solid line; men, dashed line. Partnered was married or living as married; divorced includes separated. Social network size (1–573) was a sum of responses to eight questions about the number of social contacts the respondent knows (e.g. siblings, children, colleagues, etc.), with network size increasing from smallest (Q1) to largest (Q4). Social participation was a sum of responses to eight questions about regular (≥ once per month) participation in different social activities, that was re-classified into four levels of social participation.
(DOCX)

S2 Fig Adjusted mean waist circumference (women, panel A; men, panel B) and body mass index (women, panel A; men, panel B) across levels of social participation, by living arrangement, among older women and men in the Canadian Longitudinal Study on Aging (2012–15). Social participation was a sum of responses to eight questions about regular (≥ once per month) participation in different social activities that was re-classified into four levels of social participation.
(DOCX)
