# Supplementary material for: Associations between social connections, their interactions, and obesity differ by gender: A population-based, cross-sectional analysis of the Canadian Longitudinal Study on Aging
Source: PLoS One. 2020 Jul 30;15(7):e0235977. doi: 10.1371/journal.pone.0235977 (PMC7392536; doi:10.1371/journal.pone.0235977)
Supplement: S1 Table — (DOCX) [file pone.0235977.s001.docx]

**Table S1. Descriptive characteristics across structural social ties among older adults in CLSA (2012-15).**

|  | **Sample size** | **Mean (SD) age** | **Women %** | **Highest education level %** | **Non-smoker %** | **Mean (SD) WC**  **(cm)** | **Abdominal obesity %** | **Mean (SD) BMI (kg/m^2^)** | **General obesity %** |
| --- | --- | --- | --- | --- | --- | --- | --- | --- | --- |
| Total | 28238 | 62.8 (10.2) | 50.6 | 45.7 | 31.8 | 94.2 (14.6) | 43.89 | 28.0 (5.4) | 29.24 |
| **Marital status ^*^** |  |  |  |  |  |  |  |  |  |
| Partnered | 19617 | 61.8 (9.9) | 44.2 | 48.6 | 32.4 | 94.3 (14.4) | 41.6 | 27.9 (5.1) | 28.0 |
| Single | 2404 | 59.7 (9.5) | 54.5 | 48.3 | 33.2 | 95.0 (16.0) | 46.9 | 28.6 (6.3) | 33.4 |
| Widowed | 2565 | 73.1 (8.5) | 74.2 | 29.5 | 31.5 | 93.2 (14.3) | 51.8 | 28.0 (5.5) | 30.4 |
| Divorced | 3652 | 62.8 (9.4) | 65.8 | 40.0 | 28.0 | 93.3 (15.0) | 48.4 | 28.3 (5.9) | 32.4 |
| **Living arrangement** |  |  |  |  |  |  |  |  |  |
| Co-living | 22008 | 61.6 (9.9) | 46.6 | 47.7 | 32.3 | 94.2 (14.5) | 42.3 | 28.0 (5.2) | 28.6 |
| Lone-living | 6230 | 67.1 (10.1) | 64.7 | 38.7 | 30.0 | 93.9 (15.0) | 49.4 | 28.2 (5.8) | 31.5 |
| **Social network size (quartile)** ^†^ | | |  |  |  |  |  |  |  |
| Largest, Q4 (220-573) | 7112 | 60.7(9.8) | 46.7 | 54.3 | 33.8 | 94.9 (14.7) | 44.5 | 28.41 (5.3) | 31.8 |
| Q3 (146-219) | 7025 | 61.2 (9.9) | 51.0 | 49.1 | 32.4 | 93.9 (14.7) | 42.9 | 28.1 (5.4) | 29.2 |
| Q2 (86-145) | 7025 | 63.4 (10.0) | 54.0 | 42.8 | 31.8 | 93.4 (14.4) | 43.2 | 27.8 (5.3) | 27.7 |
| Smallest, Q1 (1-85) | 7076 | 65.8 (10.3) | 50.8 | 36.5 | 29.2 | 94.4 (14.6) | 45.0 | 27.8 (5.4) | 28.3 |
| **Social participation** ^‡^ | | |  |  |  |  |  |  |  |
| A lot (5-8) | 11706 | 63.9 (10.3) | 54.5 | 53.6 | 35.6 | 93.4 (14.3) | 43.5 | 27.9 (5.2) | 28.2 |
| Some (3-4) | 11390 | 61.8 (10.0) | 49.5 | 43.9 | 30.6 | 93.9 (14.6) | 42.4 | 27.9 (5.3) | 28.5 |
| A few (1-2) | 4721 | 62.5 (10.2) | 44.7 | 32.6 | 26.2 | 96.3 (15.1) | 47.9 | 28.5 (5.8) | 32.8 |
| None (0) | 421 | 62.9 (10.4) | 40.4 | 23.5 | 23.3 | 98.5 (16.0) | 51.3 | 28.9 (6.2) | 36.6 |
| BMI, body mass index; CLSA, Canadian Longitudinal Study on Aging; WC, waist circumference. Highest education level was at least a Bachelor’s degree from a university, or higher. ^*^Partnered was married or living as married; divorced includes separated. ^†^ Social network size (1-573) was a sum of responses to eight questions about the number of social contacts the respondent knows (e.g. siblings, children, colleagues, etc), with network size increasing from smallest (Q1) to largest (Q4). ^‡^ Social participation was a sum of responses to eight questions about regular (≥ once per month) participation in different social activities, that was re-classified into four levels of social participation (0 (none), 1-2 (a few), 3-4 (some), 5-8 (a lot)). | | | | | | | | | |
