# Supplementary material for: Associations between social connections, their interactions, and obesity differ by gender: A population-based, cross-sectional analysis of the Canadian Longitudinal Study on Aging
Source: PLoS One. 2020 Jul 30;15(7):e0235977. doi: 10.1371/journal.pone.0235977 (PMC7392536; doi:10.1371/journal.pone.0235977)
Supplement: S2 Table — (DOCX) [file pone.0235977.s002.docx]

**Table S2. Independent associations between structural social ties and adiposity among older women and men in the CLSA (2012-15).**

|  | **Waist circumference (cm)** | | **Body mass index (kg/m2)** | |
| --- | --- | --- | --- | --- |
| **Social ties*** | **Women** | **Men** | **Women** | **Men** |
| **Marital status** | |  |  |  |
| Partnered | Ref | Ref | Ref | Ref |
| Single | 4.32 (3.34, 5.29) ^§^**^‖^** | 0.62 (-0.38, 1.63) **^‖^** | 1.73 (1.3, 2.16) ^§^**^‖^** | 0.01 (-0.38, 0.4) **^‖^** |
| Widowed | 1.86 (1.04, 2.68) ^§^ | 1.16 (0.05, 2.27) ^†^ | 0.85 (0.5, 1.2) ^§^**^‖^** | 0.16 (-0.25, 0.58) **^‖^** |
| Divorced | 2.18 (1.43, 2.92) ^§^**^‖^** | 0.00 (-0.84, 0.85) **^‖^** | 0.90 (0.57, 1.22) ^§^**^‖^** | -0.12 (-0.44, 0.2) **^‖^** |
| **Living arrangement** | |  |  |  |
| Co-living | Ref | Ref | Ref | Ref |
| Lone-living | 0.52 (-0.18, 1.23) **^‖^** | -1.01 (-1.79, -0.23) ^†^**^‖^** | 0.27 (-0.04, 0.57) **^‖^** | -0.7 (-1.01, -0.4) ^§^**^‖^** |
| **Social network size (quartile)** | | |  |  |
| Largest, Q4 (220-573) | Ref | Ref | Ref | Ref |
| Q3 (146-219) | -0.39 (-1.03, 0.24) | -1.06 (-1.64, -0.49) ^§^ | -0.26 (-0.53, 0.02) | -0.49 (-0.71, -0.27) ^§^ |
| Q2 (86-145) | -0.83 (-1.47, -0.2) ^†^**^‖^** | -1.77 (-2.36, -1.18) ^§^ | -0.41 (-0.68, -0.13) ^‡^**^‖^** | -0.88 (-1.1, -0.66) ^§^**^‖^** |
| Smallest, Q1 (1-85) | -0.83 (-1.5, -0.16) ^†^**^‖^** | -2.02 (-2.64, -1.4) ^§^ | -0.40 (-0.69, -0.11) ^‡^**^‖^** | -1.03 (-1.26, -0.79) ^§^**^‖^** |
| **Social participation** | | |  |  |
| A lot (5-8) | Ref | Ref | Ref | Ref |
| Some (3-4) | 0.33 (-0.15, 0.82) **^‖^** | -0.55 (-1.01, -0.08) ^†^**^‖^** | 0.03 (-0.18, 0.24) **^‖^** | 0.00 (-0.43, -0.09) ^‡^**^‖^** |
| A few (1-2) | 2.21 (1.5, 2.92) ^§^**^‖^** | 0.44 (-0.19, 1.06) **^‖^** | 0.88 (0.57, 1.2) ^§^**^‖^** | -0.05 (-0.29, 0.18) **^‖^** |
| None (0) | 4.19 (1.86, 6.52) ^§^**^‖^** | 0.89 (-1.00, 2.78) **^‖^** | 1.44 (0.43, 2.45) ^‡^**^‖^** | -0.07 (-0.79, 0.66) **^‖^** |
| BMI, body mass index; WC, waist circumference. Gender-specific coefficients (95% CI) of waist circumference and body mass index obtained by multivariable linear regression analysis of sample (N = 28238) using an interaction term between each structural social tie and male/female adjusted for age, age^2^, education, smoking, province plus the other three structural social ties. ***Partnered** was married or living as married; **divorced** includes separated. **Social network size** (1-573) was a sum of responses to eight questions about the number of social contacts the respondent knows (e.g. siblings, children, colleagues, etc), with network size increasing from smallest (Q1) to largest (Q4). **Social participation** was a sum of responses to eight questions about regular (≥ once per month) participation in different social activities, that was re-classified into four levels of social participation (0 (none), 1-2 (a few), 3-4 (some), 5-8 (a lot)). † p < 0.05; ‡ p < 0.01; § p < 0.001; ‖ p for interaction < 0.05. | | | | |
