# Supplementary material for: Associations between social connections, their interactions, and obesity differ by gender: A population-based, cross-sectional analysis of the Canadian Longitudinal Study on Aging
Source: PLoS One. 2020 Jul 30;15(7):e0235977. doi: 10.1371/journal.pone.0235977 (PMC7392536; doi:10.1371/journal.pone.0235977)
Supplement: S3 Table — (DOCX) [file pone.0235977.s003.docx]

**S3 Table. Independent associations between marital status and adiposity, by living arrangement, among older women and men in the CLSA (2012-15).**

|  | **Waist circumference (cm)** | | **Body mass index (kg/m^2^)** | |
| --- | --- | --- | --- | --- |
|  | **Co-living** | **Lone-living** | **Co-living** | **Lone-living** |
| **Women (n=14,289)** |  |  |  |  |
| Partnered* | Ref | Ref | Ref | Ref |
| Single | 4.17 (2.59,5.75) | 2.01 (-0.95-4.97) | 1.65 (0.95,2.36) | 0.79 (-0.56,2.14) |
| Widowed | 3.57 (2.26,4.88) † | -0.97 (-3.85,1.91) † | 1.11 (0.53,1.69) † | -0.4 (-1.71,0.92) † |
| Divorced/separated | 2.13 (1.13,3.14) | -0.21 (-3.09,2.67) | 0.93 (0.48,1.38) | -0.2 (-1.51,1.12) |
| **Men (n=13,949)** |  |  |  |  |
| Partnered* | Ref | Ref | Ref | Ref |
| Single | -0.26 (-1.88,1.36) | -0.05 (2.76,2.65) | -0.16 (-0.78,0.45) | -0.22 (-1.13,0.69) |
| Widowed | 0.34 (-1.81,2.48) | 0.35 (-2.36,3.05) | 0.10 (-0.71,0.9) | 0.01 (-0.89,0.92) |
| Divorced/separated | 0.28 (-0.97,1.53) | -1.09 (-3.72,1.55) | 0.01 (-0.44,0.47) | -0.43 (-1.31,0.45) |
| CLSA, Canadian Longitudinal Study on Aging. Gender-specific coefficients (CI95) of waist circumference and body mass index associated with marital status by living arrangement. Sex-stratified models included interaction term between marital status and living arrangement adjusted for age, age^2^, education, smoking, province, social network size and social participation. ^*^ Partnered was married or living as married. †p-interaction< 0.05. | | | | |
