# Supplementary material for: Associations between social connections, their interactions, and obesity differ by gender: A population-based, cross-sectional analysis of the Canadian Longitudinal Study on Aging
Source: PLoS One. 2020 Jul 30;15(7):e0235977. doi: 10.1371/journal.pone.0235977 (PMC7392536; doi:10.1371/journal.pone.0235977)
Supplement: S4 Table — (DOCX) [file pone.0235977.s004.docx]

**S4 Table. Independent association between marital status and adiposity, by social participation or by social network size, among older adults in CLSA (2012-15).**

|  | **Waist circumference (cm)** | | **Body mass index (kg/m^2^)** | |
| --- | --- | --- | --- | --- |
|  | Social participation^*^ | Social network size^†^ | Social participation | Social network size |
| **Women (n= 14,289)** ^‡^ | |  |  |  |
| Partnered | Ref | Ref | Ref | Ref |
| Single | 5.16 (2.91,7.41) | 3.95 (2.21,5.68) | 1.89 (0.89,2.9) | 1.44 (0.67,2.21) |
| Widowed | 1.61 (-0.25,3.47) | 1.64 (0.22,3.05) | 0.62 (-0.21,1.46) | 0.58 (-0.03,1.19) |
| Divorced | 3.72 (1.95,5.48) | 3.27 (1.88,4.66) | 1.54 (0.76,2.32) ^§^ | 1.11 (0.49,1.73) |
| **Men (n= 13,949)** ^‡^ | |  |  |  |
| Partnered | Ref | Ref | Ref | Ref |
| Single | -0.76 (-2.93,1.4) | -0.66 (-2.45,1.13) | -0.40 (-1.23,0.43) | -0.47 (-1.15,0.21) |
| Widowed | 1.76 (-0.8,4.32) | 0.93 (-1.08,2.93) | 0.51 (-0.49,1.5) | 0.30 (-0.44,1.04) |
| Divorced | -0.54 (-2.46,1.38) | 0.13 (-1.52,1.77) | -0.26 (-0.96,0.44) | 0.03 (-0.57,0.62) |
| CLSA, Canadian Longitudinal Study on Aging. Gender-specific coefficients (CI95) of waist circumference and body mass index and marital status at the level of no social participation or 1 social contact. Sex-stratified models adjusted for age, age^2^, education, smoking, province, marital status. ^*^Partnered was married or living as married; divorced includes separated. †Social network size (1-573) was a sum of responses to eight questions about the number of social contacts the respondent knows (e.g. siblings, children, colleagues, etc). ^‡^Social participation was a sum of responses to eight questions about regular (≥ once per month) participation in different social activities. The coefficients presented in this table represent the main effect of marital status on each outcome at 0 social participation/network. ^§^p-interaction< 0.05. | | | | |
