# Supplementary material for: Associations between social connections, their interactions, and obesity differ by gender: A population-based, cross-sectional analysis of the Canadian Longitudinal Study on Aging
Source: PLoS One. 2020 Jul 30;15(7):e0235977. doi: 10.1371/journal.pone.0235977 (PMC7392536; doi:10.1371/journal.pone.0235977)
Supplement: S5 Table — (DOCX) [file pone.0235977.s005.docx]

**S5 Table. Independent association between lack of social participation and adiposity, by average social network size, among older women and men in the CLSA (2012-15).**

|  | **Social network size (mean)^*^** | |
| --- | --- | --- |
|  | **Waist circumference (cm)** | **Body mass index (kg/m2)** |
| **Women**  0 regular social activities† (n=14,289) | -0.63 (-0.88, -0.38) ^‡^ | -0.28 (-0.39, -0.17) ^‡^ |
| **Men**  0 regular social activities† (n=13,949) | -0.27 (-0.51, -0.03) | -0.06 (-0.15, 0.04) |
| Gender-specific coefficients (CI95) of body mass index and waist circumference with social network size (mean) at 0 social participation. The coefficients presented in this table represent the main effect of social network size on each outcome at 0 social participation. Sex-stratified models included interaction term between social network size and social participation adjusted for age, age^2^, education, smoking, province, marital status and living arrangement. ^*^ Social network size (1-573) was a sum of responses to eight questions about the number of social contacts the respondent knows (e.g. siblings, children, colleagues, etc). ^†^ Social participation was a sum of responses to eight questions about regular (≥ once per month) participation in different social activities. ^‡^ p-interaction< 0.05. | | |
