# Supplementary material for: Associations between social connections, their interactions, and obesity differ by gender: A population-based, cross-sectional analysis of the Canadian Longitudinal Study on Aging
Source: PLoS One. 2020 Jul 30;15(7):e0235977. doi: 10.1371/journal.pone.0235977 (PMC7392536; doi:10.1371/journal.pone.0235977)
Supplement: S6 Table — (DOCX) [file pone.0235977.s006.docx]

**S6 Table** **Independent association between living arrangement and adiposity, by social participation and social network size, among older women and men in CLSA (2012-15).**

|  | **Waist circumference**  **(cm)** | | **Body mass index**  **(kg/m^2^)** | |
| --- | --- | --- | --- | --- |
|  | Social participation^*^ | Social network size^†^ | Social participation | Social network size |
| **Women (n=14,289)** |  |  |  |  |
| Co-living | Ref | Ref | Ref | Ref |
| Lone-living | -0.13 (-1.61,1.35) | -0.14 (-1.29,1.00) | -0.08 (-0.74,0.59) | -0.27 (-0.78,0.23) |
| **Men (n=13,949)** |  |  |  |  |
| Co-living | Ref | Ref | Ref | Ref |
| Lone-living | 0.66 (-0.96,2.28) | 0.96 (-0.4,2.33) | 0.13 (-0.48,0.74) | 0.10 (-0.4,0.61) |
| CLSA, Canadian Longitudinal Study on Aging. Gender-specific coefficients (CI95) of waist circumference and body mass index associated with living arrangement at 0 social participation or 1 social contact. The coefficients presented in this table represent the main effect of living arrangement on each outcome at 0 social participation/network Sex-stratified models included interaction term between living arrangement and social participation/network size adjusted for age, age^2^, education, smoking, province and marital status. ^*^ Social participation was a sum of responses to eight questions about regular (≥ once per month) participation in different social activities. ^†^ Social network size (1-573) was a sum of responses to eight questions about the number of social contacts the respondent knows (e.g. siblings, children, colleagues, etc). | | | | |
