# Supplementary material for: Associations between social connections, their interactions, and obesity differ by gender: A population-based, cross-sectional analysis of the Canadian Longitudinal Study on Aging
Source: PLoS One. 2020 Jul 30;15(7):e0235977. doi: 10.1371/journal.pone.0235977 (PMC7392536; doi:10.1371/journal.pone.0235977)
Supplement: S1 Fig — Adjusted mean body mass index with A, marital status, B, living arrangement, C, social participation and D, social network size among older women and men in the Canadian Longitudinal Study on Aging CLSA (2012–15). Women, solid line; men, dashed line. Partnered was married or living as married; divorced includes separated. Social network size (1–573) was a sum of responses to eight questions about the number of social contacts the respondent knows (e.g. siblings, children, colleagues, etc.), with network size increasing from smallest (Q1) to largest (Q4). Social participation was a sum of responses to eight questions about regular (≥ once per month) participation in different social activities, that was re-classified into four levels of social participation. (DOCX) [file pone.0235977.s007.docx]

| **A**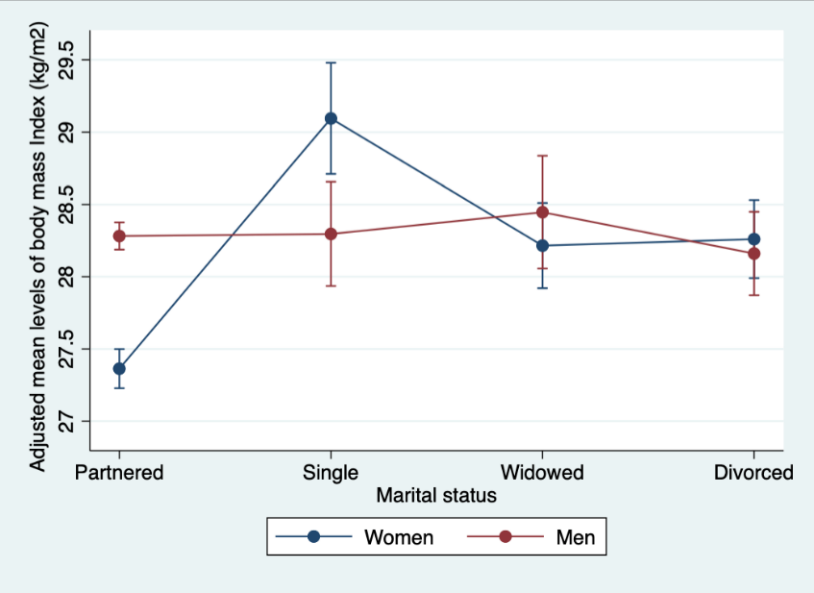 | **C**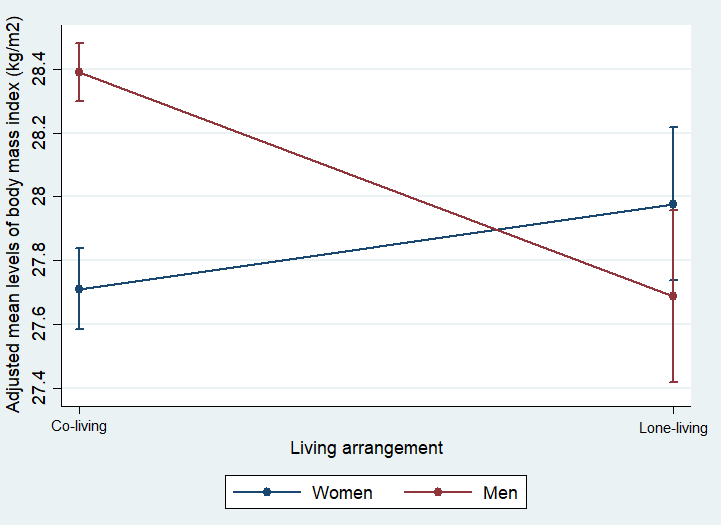 |
| --- | --- |
| **B**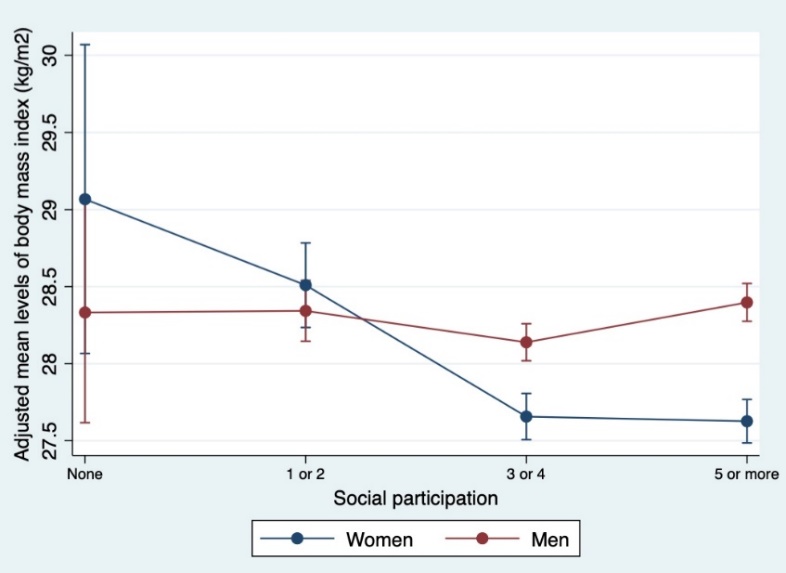 | **D**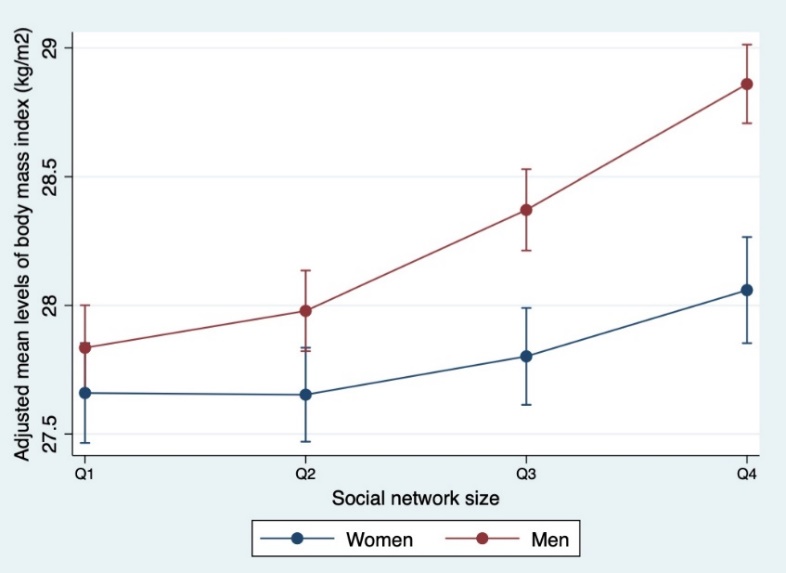 |
| **S1 Fig. Adjusted mean body mass index with A, marital status, B, living arrangement, C, social participation and D, social network size among older women and men in the Canadian Longitudinal Study on Aging CLSA (2012-15).** Women, solid line; men, dashed line. Partnered was married or living as married; divorced includes separated. Social network size (1-573) was a sum of responses to eight questions about the number of social contacts the respondent knows (e.g. siblings, children, colleagues, etc.), with network size increasing from smallest (Q1) to largest (Q4). Social participation was a sum of responses to eight questions about regular (≥ once per month) participation in different social activities, that was re-classified into four levels of social participation (0 (none), 1-2 (a few), 3-4 (some), 5-8 (a lot)). | |
