# Supplementary material for: Associations between social connections, their interactions, and obesity differ by gender: A population-based, cross-sectional analysis of the Canadian Longitudinal Study on Aging
Source: PLoS One. 2020 Jul 30;15(7):e0235977. doi: 10.1371/journal.pone.0235977 (PMC7392536; doi:10.1371/journal.pone.0235977)
Supplement: S2 Fig — Adjusted mean waist circumference (women, panel A; men, panel B) and body mass index (women, panel A; men, panel B) across levels of social participation, by living arrangement, among older women and men in the Canadian Longitudinal Study on Aging (2012–15). Social participation was a sum of responses to eight questions about regular (≥ once per month) participation in different social activities that was re-classified into four levels of social participation. (DOCX) [file pone.0235977.s008.docx]

| A 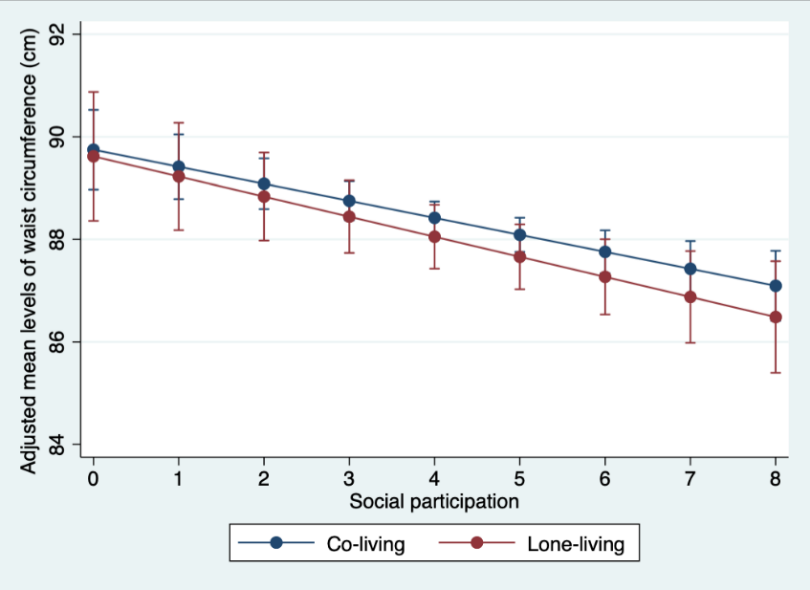 | C 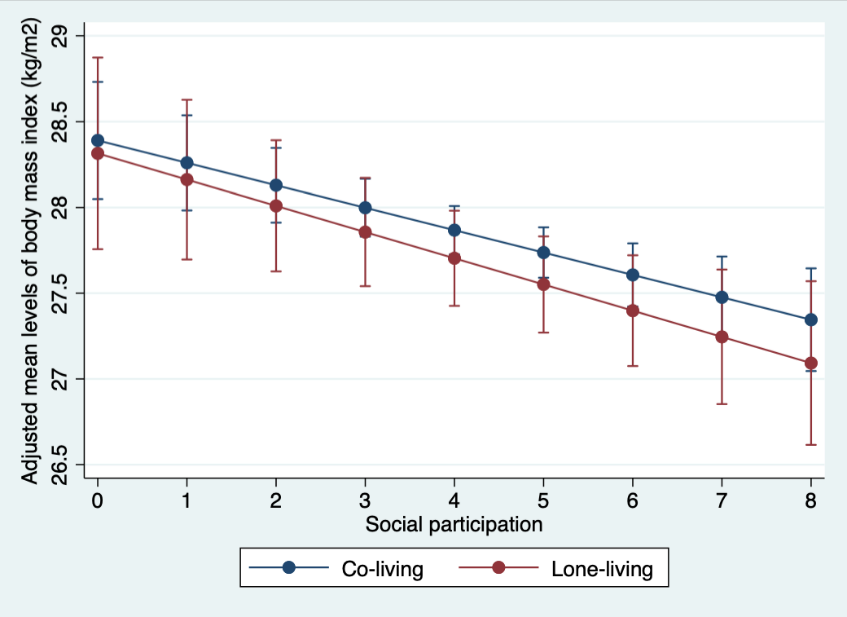 |
| --- | --- |
| B 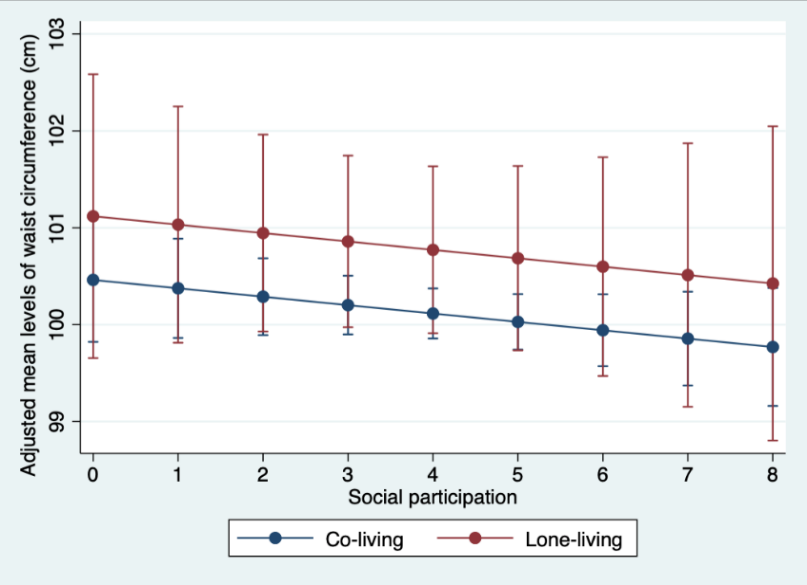 | D 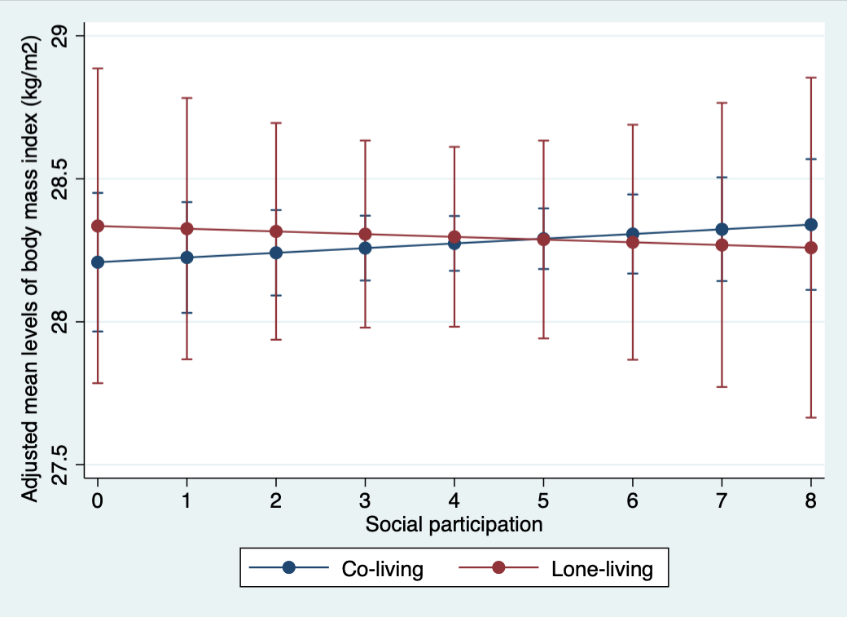 |
| **S2 Fig. Adjusted mean waist circumference (women, panel A; men, panel B) and body mass index (women, panel A; men, panel B) across levels of social participation, by living arrangement, among older women and men in the Canadian Longitudinal Study on Aging (2012-15).** Social participation was a sum of responses to eight questions about regular (≥ once per month) participation in different social activities. | |
